# Functional and Computational Analysis of Amino Acid Patterns Predictive of Type III Secretion System Substrates in *Pseudomonas syringae*

**DOI:** 10.1371/journal.pone.0036038

**Published:** 2012-04-27

**Authors:** Lisa M. Schechter, Joy C. Valenta, David J. Schneider, Alan Collmer, Eric Sakk

**Affiliations:** 1 Department of Biology, University of Missouri-St. Louis, St. Louis, Missouri, United States of America; 2 Plant-Microbe Interactions Research Unit, Robert W. Holley Center for Agriculture and Health, Agricultural Research Service, United States Department of Agriculture, Ithaca, New York, United States of America; 3 Department of Plant Pathology and Plant-Microbe Biology, Cornell University, Ithaca, New York, United States of America; 4 Department of Computer Science, Morgan State University, Baltimore, Maryland, United States of America; University of Wisconsin-Milwaukee, United States of America

## Abstract

Bacterial type III secretion systems (T3SSs) deliver proteins called effectors into eukaryotic cells. Although N-terminal amino acid sequences are required for translocation, the mechanism of substrate recognition by the T3SS is unknown. Almost all actively deployed T3SS substrates in the plant pathogen *Pseudomonas syringae* pathovar tomato strain DC3000 possess characteristic patterns, including (i) greater than 10% serine within the first 50 amino acids, (ii) an aliphatic residue or proline at position 3 or 4, and (iii) a lack of acidic amino acids within the first 12 residues. Here, the functional significance of the *P. syringae* T3SS substrate compositional patterns was tested. A mutant AvrPto effector protein lacking all three patterns was secreted into culture and translocated into plant cells, suggesting that the compositional characteristics are not absolutely required for T3SS targeting and that other recognition mechanisms exist. To further analyze the unique properties of T3SS targeting signals, we developed a computational algorithm called TEREE (Type III Effector Relative Entropy Evaluation) that distinguishes DC3000 T3SS substrates from other proteins with a high sensitivity and specificity. Although TEREE did not efficiently identify T3SS substrates in *Salmonella enterica*, it was effective in another *P. syringae* strain and *Ralstonia solanacearum*. Thus, the TEREE algorithm may be a useful tool for identifying new effector genes in plant pathogens. The nature of T3SS targeting signals was additionally investigated by analyzing the N-terminus of FtsX, a putative membrane protein that was classified as a T3SS substrate by TEREE. Although the first 50 amino acids of FtsX were unable to target a reporter protein to the T3SS, an AvrPto protein substituted with the first 12 amino acids of FtsX was translocated into plant cells. These results show that the T3SS targeting signals are highly mutable and that secretion may be directed by multiple features of substrates.

## Introduction

Gram-negative bacteria have developed a wide variety of mechanisms to export proteins. One of the best studied protein secretion devices is the type III secretion system (T3SS), which transports extracellular components of the flagellum [1]. Some Gram-negative pathogens and symbionts also contain T3SSs that deliver proteins called effectors directly from the bacterial cytoplasm into host cells during infection [2]. Recent findings suggest that T3SSs may additionally translocate extracellular bacterial proteins into host cells [3], [4]. Once inside the host cell cytoplasm, effectors mimic host proteins and manipulate signaling pathways to promote bacterial survival and growth during infection [5], [6].

Identifying the complete collection of T3SS effectors produced by a particular bacterium has proven difficult for several reasons. First, many effectors have similar or redundant functions inside host cells, which may mask phenotypes in screens for less virulent mutants. Studies in *Salmonella enterica* and *Pseudomonas syringae* have shown that deletion of multiple effector genes is often required to observe attenuation in virulence assays [7]–[9]. Second, genetic screens to identify new effectors are often labor intensive [10]–[13]. Proteomic analysis of culture supernatants may be a more efficient way to identify T3SS substrates [14], [15]. However, this method may fail to discover effectors that are secreted in small amounts or are only deployed upon host cell contact. Finally, many effectors appear to be unique to certain species or even strains of bacteria. Thus, homology searches have only been successful at identifying a subset of the effectors present in any one bacterium.

Understanding how effector proteins are targeted for secretion is crucial for discovering all of the effector genes in bacteria, as well as for developing new methods to inhibit T3SS function. Although the mechanism of substrate recognition by the T3SS is unclear, two models have been proposed to explain how effectors are distinguished from other bacterial proteins. In the first model, effectors are targeted to the T3SS by N-terminal amino acid sequences. This model is based on studies showing that the first ∼15 amino acids of the *Yersinia* effector YopE are essential for secretion into the extracellular milieu [16], [17]. A larger region (∼50 N-terminal amino acids) is required for effector translocation into host cells [16], [17]. The additional sequences required for efficient translocation may be involved in mediating the delivery of effectors from an extracellular location into host cells [3].

In the second model of T3SS substrate recognition, sequences within the first 15 codons of mRNAs form secondary structures that target effector proteins for cotranslational export through the T3SS [18]. In support of this hypothesis, frameshift mutations that drastically change the N-terminal amino acid sequences of effector proteins but minimally alter the mRNA sequence do not abrogate effector secretion or translocation by the T3SS [18]–[21]. However, effector secretion is also unaffected by synonymous changes within the first 15 codons that considerably affect the mRNA secondary structure without altering the protein sequence [21], [22]. The observation that effectors are deployed in the presence of translation inhibitors additionally casts doubt on the cotranslational secretion theory [22]. Altogether, these findings indicate that the T3SS targeting signal within the N-terminal 15 amino acids of effectors is highly degenerate and tolerant of mutations. Thus, it may be impossible to identify a consensus T3SS recognition sequence within effector proteins.

In addition to endogenous targeting signals, effectors may be guided to the T3SS by accessory factors called chaperones. T3SS chaperones are small, usually acidic proteins that have similar structures, even though their amino acid sequences are not significantly similar. Chaperone genes are generally encoded adjacent to effector genes, or within T3SS gene clusters. They bind to the effector chaperone-binding domain (CBD), a ∼50–100 amino acid region that is directly downstream from the N-terminal secretion targeting signal [2]. Although many chaperones are dedicated to binding only one effector, some chaperones are promiscuous and bind to several different effectors [23]. Two lines of evidence support a role for chaperones in effector targeting. First, deletion or mutation of the CBDs in the *Salmonella* effectors SopA, SopE, SptP, and SipA causes these proteins to be secreted into culture via the flagellar export pathway, rather than the *Salmonella* pathogenicity island 1 (SPI-1)-encoded T3SS [24]–[26]. This finding indicates that at least some effectors require chaperones for targeting to the proper T3SS. Second, chaperones can interact with proteins at the base of the T3SS in *Salmonella*, enteropathogenic *E. coli*, and *Chlamydia* [27]–[29]. However, in certain situations YopE from *Yersinia* does not require its dedicated chaperone, SycE (YerA), for T3SS-mediated secretion or translocation [17], [30], [31]. Thus, the N-terminal 15 amino acids of effectors are sufficient for T3SS targeting, and chaperones may serve to enhance the process.

Although the molecular mechanisms that underlie effector targeting to the T3SS remain obscure, several structural, bioinformatic, and computational analyses indicate that the N-termini of effector proteins possess common features, including: (i) flexibility and disorder in solution [32], [33], (ii) amphipathicity [22], [34], [35], and (iii) bias for particular amino acids [35]–[38]. In fact, the N-terminal amino acid sequences of actively deployed effectors in *P. syringae* pathovar tomato (*P. s.* tomato) strain DC3000 have been examined extensively and generally contain three patterns. First, DC3000 effector N-termini are enriched in polar amino acids, especially serine [11], [37], [39]. Second, DC3000 effectors usually contain an aliphatic amino acid or proline at the third or fourth position [39]–[41]. Finally, DC3000 T3SS substrates also generally lack negatively charged amino acids within the first 12 residues [39]–[41]. These characteristics have been successfully used for their predictive value as part of a bioinformatic workflow for identifying candidate effectors in *P. syringae* genomes [41], [42].

The targeting patterns in *P. syringae* effectors are also found in flagellar secretion substrates and in a subset of T3SS effectors in other plant and animal pathogens. For example, the *Yersinia* effector YopE possesses the three major patterns, including an unusually high serine content of 28% in the first 50 residues. However, many T3SS substrates from animal pathogens lack one or more of these characteristic patterns [43]. This observation suggests that the characteristic targeting patterns of *P. syringae* effectors may not mediate secretion, or that two or more classes of effectors exist in bacteria with quite different N-terminal amino acid patterns.

In this study, we sought to better understand how T3SS substrates are distinguished from other proteins in *P. s.* tomato DC3000, a model pathogen of the important crop tomato and the model plant Arabidopsis. This organism is an ideal subject for bioinformatic and computational studies on T3SS targeting signals because its genome sequence has been determined and it encodes over 50 experimentally validated T3SS substrates [41], [44]–[46]. We first analyzed whether the characteristic targeting patterns found in most DC3000 effectors are required for the T3SS-dependent secretion of AvrPto. An altered AvrPto protein lacking all of the patterns was targeted for secretion as well as wild-type AvrPto. To determine whether DC3000 effectors have other distinctive properties, we developed a computational algorithm that measures differences between the amino acid sequences of T3SS substrates and nonsecreted proteins. In contrast to other computational T3SS substrate prediction models that utilize Naïve Bayesian, artificial neural network (ANN), or support vector machine (SVM) classification algorithms, our method is based on an information theory approach. The performance of our algorithm was analyzed in *P. syringae* and other bacteria with T3SSs, and in comparison to other T3SS prediction models. We show that our computational algorithm is a useful tool for recognizing T3SS substrates in three plant pathogens.

## Results

### Examination of T3SS targeting patterns in the *P. syringae* AvrPto effector protein

**Figure 1:**
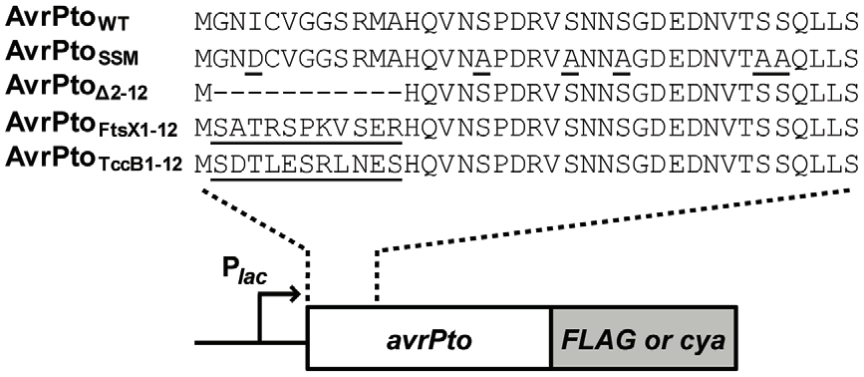
Schematic diagram of AvrPto mutants examined in this study. Plasmids were constructed that express wild-type or mutant versions of the *avrPto* gene fused in frame to either *FLAG* epitope tag sequences or *cya* (calmodulin-dependent adenylate cyclase). Each gene was expressed from an upstream *lac* promoter (P*_lac_*). The sequences of the first 50 amino acids of each protein are shown above the *avrPto* gene. Amino acids in the mutant proteins that differ from the wild-type AvrPto sequence are underlined. Dashes within the AvrPto_Δ2–12_ sequence indicate deleted residues.

Despite the value of the characteristic T3SS targeting patterns in predicting high-probability *P. syringae* effector candidates, the importance of these sequences in mediating secretion has not been examined. We therefore analyzed the significance of the targeting patterns in AvrPto, a well-studied *P. syringae* effector that suppresses plant immune responses triggered by pathogen-associated molecular patterns (PAMPs) [47]. A previous study showed that the first ∼50 amino acids of AvrPto are required for efficient secretion into culture and translocation into plant cells by the *P. s.* tomato DC3000 T3SS [40]. Plasmids were constructed that express C-terminally FLAG epitope-tagged wild-type or secretion signal mutant versions of AvrPto (AvrPto_WT_ and AvrPto_SSM_, respectively). AvrPto_SSM_ contains several mutations. The fourth residue (isoleucine) is substituted with aspartate, and most of the serines within the first 50 amino acids are changed to alanine (Figure 1). The mutant thus lacks all three of the *P. syringae* characteristic T3SS targeting patterns.

**Figure 2:**
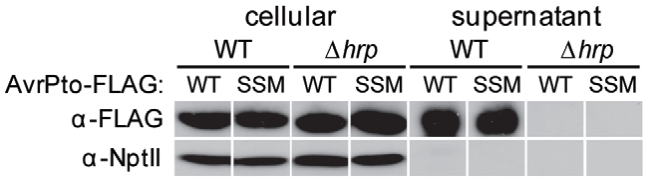
Secretion of AvrPto_WT_ and AvrPto_SSM_ by DC3000. Wild-type and T3SS mutant (Δ*hrp*) DC3000 strains containing plasmids that express AvrPto_WT_ or AvrPto_SSM_ were grown in *hrp*-derepressing fructose minimal medium (HDM). Cultures were separated into cellular and supernatant fractions by centrifugation and filtration, and an immunoblot analysis was performed after electrophoresis of protein samples through a 12.5% SDS–PAGE gel. The supernatant samples are 15-fold more concentrated than the cellular samples. The 21 kDa AvrPto_WT_ and AvrPto_SSM_ proteins were detected using primary antibodies against the FLAG epitope. The NptII protein (29.1 kDa) expressed from pUFR034 was also detected as a cytoplasmic control using primary antibodies against NptII. The results shown were taken from samples collected during a single experiment. Similar results were observed in an independently conducted experiment.

The two plasmids expressing AvrPto_WT_ or AvrPto_SSM_ were transferred into wild-type DC3000 and a Δ*hrp* mutant derivative, which lacks the entire T3SS coding region [48]. These strains were grown in *hrp*-derepressing minimal medium (HDM) to induce T3SS gene expression, and cellular and supernatant protein samples were collected. Approximately equal levels of AvrPto_SSM_ and AvrPto_WT_ were isolated from the culture supernatants of wild-type DC3000 (Figure 2). In addition, secretion of both AvrPto_SSM_ and AvrPto_WT_ was dependent on an intact T3SS. As a control, we examined the location of neomycin phosphotransferase II (NptII), a cytoplasmic protein. NptII was detected in bacterial cells but not culture supernatants, showing that cytoplasmic proteins did not leak into the growth medium during the experiment (Figure 2). Overall, these results show that the characteristic targeting patterns of *P. syringae* T3SS substrates are not required for the secretion of AvrPto.

**Figure 3:**
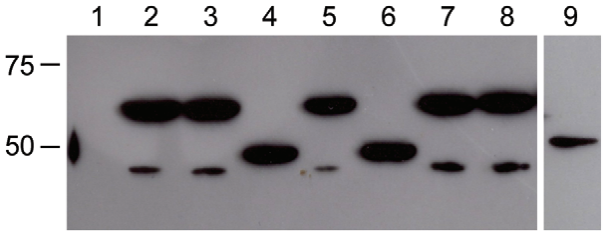
Expression of AvrPto-Cya hybrid proteins in *P. s.* tomato DC3000. DC3000 strains containing plasmids that express Cya fusion proteins were grown in culture and protein samples were separated in a 12.5% SDS–PAGE gel. An immunoblot analysis was performed using primary antibodies against Cya. The protein in each lane and its estimated molecular weight is: Lane 1, empty vector; lane 2, AvrPto_Δ2–12_-Cya (60.9 kDa); lane 3, AvrPto_WT(1–164)_-Cya (62.0 kDa); lane 4, AvrPto_WT(1–50)_-Cya (48.9 kDa); lane 5, AvrPto_SSM(1–164)_-Cya (61.9 kDa); lane 6, AvrPto_SSM(1–50)_-Cya (48.9 kDa); lane 7, AvrPto_FtsX(1–12)_-Cya (62.1 kDa); lane 8, AvrPto_TccB(1–12)_-Cya (62.2 kDa); lane 9, FtsX_1–50_-Cya (50.8 kDa). The positions of protein standards on the gel are indicated to the left of the blot.

Although the secretion signal mutations did not affect AvrPto export into the extracellular milieu, we suspected that they might reduce AvrPto translocation into plant cells. In a previous study, we showed that *P. s.* tomato DC3000 efficiently translocates an AvrPto-Cya hybrid protein into the leaves of tomato or *Nicotiana benthamiana* plants in a T3SS-dependent manner [40]. Cya is a bacterial adenylate cyclase that produces cAMP only when it is delivered into the cytoplasm of eukaryotic cells, where it can bind to its cofactor calmodulin [16]. To test whether the characteristic effector targeting patterns are required for translocation of AvrPto, we constructed four plasmids that express different versions of *avrPto-cya* (Figure 1). Two of these plasmids express Cya hybrid proteins that include the entire 164 amino acids of AvrPto_WT_ or AvrPto_SSM_. The other two plasmids express the first 50 amino acids of AvrPto_WT_ or AvrPto_SSM_ fused to Cya. These smaller hybrid proteins were constructed because we hypothesized that the SSM mutations might have a stronger effect in the context of the minimal AvrPto translocation signal. Expression of the appropriate sized proteins in DC3000 was confirmed by immunoblot analysis (Figure 3). Smaller protein bands were detected by the anti-Cya antibodies in some lanes of the immunoblot, as has been observed in previous studies [40], [41]. These species may result from processing of the Cya hybrid protein.

To analyze AvrPto translocation, accumulation of cAMP was measured in *N. benthamiana* leaves after inoculation with wild-type or Δ*hrp* DC3000 strains expressing the various AvrPto-Cya hybrid proteins. Similar levels of cAMP were detected in *N. benthamiana* leaves inoculated with wild-type DC3000 expressing AvrPto_WT(1–164)_-Cya or AvrPto_SSM(1–164)_-Cya (Table 1). *N. benthamiana* leaves inoculated with DC3000 strains expressing AvrPto_WT(1–50)_-Cya or AvrPto_SSM(1–50)_-Cya also produced nearly the same levels of cAMP. Translocation was dependent on the T3SS, since little cAMP accumulation occurred when plant leaves were inoculated with DC3000 Δ*hrp* mutants expressing the hybrid proteins. Thus, despite lacking the common T3SS secretion signal targeting patterns, the AvrPto_SSM_ mutant was translocated into cells as well as AvrPto_WT_.

**Table 1:** Translocation of AvrPto-Cya hybrid proteins into *N. benthamiana* by *P. s.* tomato DC3000.

Cya fusion protein	Translocation by DC3000 (pmol cAMP/µg protein)	
	Wild-type	Δ*hrp*
AvrPto_WT(1–164)_	139.3±5.5	0.2±0.2
AvrPto_WT(1–50)_	112.6±14.2	0.6±0.2
AvrPto_SSM(1–164)_	152.3±15.1	0.8±0.6
AvrPto_SSM(1–50)_	124.4±19.3	0.8±0.2

### Amino acid composition comparisons between T3SS substrates and other DC3000 proteins

The characteristic targeting patterns in *P. syringae* effectors were initially identified by manual examination of amino acid sequences. We reasoned that a computational approach would more comprehensively determine properties that are unique to T3SS substrates. To begin our analysis, a substrate training set was constructed that contained most of the experimentally confirmed DC3000 T3SS substrates, which came to 38 proteins in total (Table 2). HopP1, HopAO1, HopT1-2, HopAA1-2, and HopAM1-2 were excluded from the substrate training set because they are highly homologous to other DC3000 effector proteins and thus might bias results. Several other validated effectors were also omitted because the genes that encode them in DC3000 are not expressed or are interrupted by transposons [41]. The rest of the proteins encoded in the DC3000 genome (∼5600) were used as a background data set for comparison. It is important to note that the background data set could contain T3SS substrates that have not yet been identified.

**Table 2:** DC3000 T3SS substrates and their scores after analysis by the TEREE algorithm.

Substrate^a^	Score	Amino acid sequence used to construct the T3SS substrate training set
HrpA1	−19	MVAFAGLTSKLTNLGNSAVGGVGGALQGVNTVASNATLQKNILLGTGDSL
HrpH1	−14	MPAVAFPVSSPRLLARAVQIAVLAMGALCVGCQSVDYSPPRQDRPPRLVS
HrpJ1	−27	MKIVAPPIMRILPVAPTRVVTPAAQPLPNADLHNSGTSPQQVSRFAAALI
HrpK1	−15	MRISSSPFVIVNQPTPGELALAVESPLAKALPTPVGGGGQAGVQFGQPAG
HrpW1	−29	MSIGITPRPQQTTTPLDFSALSGKSPQPNTFGEQNTQQAIDPSALLFGSD
HrpZ1	−27	MQALNSISSLQTSASLFPVSLNSDVSANTSTSSKELKAVIDQLVQALTQS
HopAK1	−31	MNTINRNIYPVSGISAQDAPVQTDQLQPQGQGIRPGHNSNLIDFGLIQQA
AvrE1	−25	MQSPSIHRNTGSIIQPTVTPDARAATDLQERAEQPRQRSSHSLSSVGKRA
AvrPto1	−35	MGNICVGGSRMAHQVNSPDRVSNNSGDEDNVTSSQLLSVRHQLAESAGLP
HopA1	−15	MNPIQSRFSSVQELRRSNVDIPALKANGQLEVDGKRYEIRAADDGTISVL
HopB1	−33	MRPVGGPAPGYYPPTYEAERPTAQAAGNDRARSSQASSSPAASVAPETPM
HopC1	−40	MTIVSGHIGKHPSLTTVQAGSSASVENQMPDPAQFSDGRWKKLPTQLSSI
HopD*	−31	
HopD1	−35	MNPLRSIQHNIATPPISGGQPLDAVGPQAQQSHPKRISPSQLSQSAHQAL
HopE1	−27	MNRVSGSSSATWQAVNDLVEQVSERTTLSTTGYQTAMGRLNKPEKSDADA
HopF2	−39	MGNICGTSGSRHVYSPSHTQRITSAPSTSTHVGGDTLTSIHQLSHSQREQ
HopG1	−21	MQIKNSHLYSASRMVQNTFNASPKMEVTNAIAKNNEPAALSATQTAKTHE
HopH1	−19	MITPSRYPGIYIAPLSNEPTAAHTFKEQAEEALDHISAAPSGDKLLRKIS
HopI1	−24	MINLTHIASSLARAALSDSTKPKMERAINVASHIAGKVALQVTSSLLEQK
HopK1	−35	MNRISTSSVNSSFNYTAPTEEAQNRFASAPDNSPLVVTTTSIAQASEGLQ
HopM1	−37	MISSRIGGAGGVKLSRVNQQHDTVPAQTAHPNAVTAGMNPPLTPDQSGSH
HopN1	−23	MYIQQSGAQSGVAAKTQHDKPSSLSGLAPGSSDAFARFHPEKAGAFVPLE
HopO1-1	−47	MGNICGTSGSNHVYSPPISPQHASGSSTPVPSASGTMLSLSHEQILSQNY
HopO1-2	−36	MNISPVSGAHGSSYPSAQSTASTASKGPSGSFLKQLGGCFSPCLGSSSTG
HopO1-3*	−29	
HopP1*	−22	
HopQ1-1	−34	MHRPITAGHTTSRLILDQSKQISRTPSESSAQSALSQQASMSSPVLERSK
HopR1	−23	MVKVTSSGFTANPLSHHADSVSPANSPPQLPEPVHLVDLSESSRKGGMRN
HopS1*	−19	
HopS2	−17	MKKSGAGTQAYALFASATGSSSKGVLSTIARHLTGCFAPNKTALHSATAV
HopT1-1	−31	MKTVSNHSIPSTNLVVDAGTETSAQKSQPVCSEIQRNSKIEKAVIEHIAD
HopT1-2*	−26	
HopU1	−23	MNINRQLPVSGSERLLTPDVGVSRQACSERHYSTGQDRHDFYRFAARLHV
HopV1	−22	MRFDAARGQKPKAPMDAPSSLRLRAIAGGMPSEEAGTTAPADVNQPPPAD
HopX1	−34	MKIHNAGLTPPLPGISNGNVGKAAQSSITQPQSQQGSYGLPPESSETRPD
HopY1	−25	MNITPLTSAAGKGSSAQGTDKISIPNSTRMINAASIKWLNKVRSAISDHI
HopAA1-1	−33	MHINRRVQQPPVTATDSFRTASDASLASSSVRSVSSDQQREINAIADYLT
HopAA1-2	−29	
HopAB2	−43	MAGINRAGPSGAYFVGHTDPEPVSGQAHGSGSGASSSNSPQVQPRPSNTP
HopAD1	−26	MLIGHSLHHMRPTAVDSSLPTSATSQTISNTKSRLDPHRVRELTFIGVGS
HopAF1	−43	MGLCISKHSGSSYSYSDSDRWQVPACPPNARSVSSHQTASASDIASGDVD
HopAG*	−15	
HopAH1	−39	MSMNTSVSNNGPVWSPVSSGNHAPSPDFSGKSSSNAVHFLSPESAHRSPS
HopAH2-1*	−19	
HopAH2-2*	+2	
HopAI1	−11	MLALKLNTSIAQAPLKKNAEAELRHMNHAEVRAHTPTRFTLNHRAPTYEV
HopAM1-1	−38	MHANPLSSFNRAQHGNLTNVEASQVKSAGTSSTTNIDSKNIEEHVADRLS
HopAM1-2*	−38	
HopAO1*	−29	
HopAQ1*	−33	
HopAS1*	−25	
PSPTO_0907*	−21	

a Experimentally validated T3SS substrates that were not included in the positive training set are denoted by asterisks.

**Figure 4:**
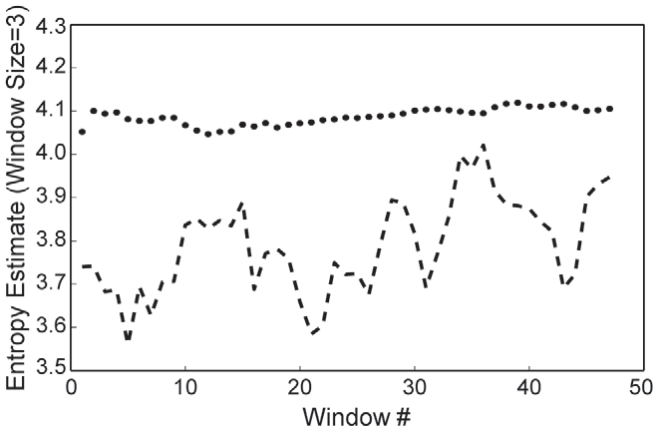
Entropy estimates for the N-terminal regions of DC3000 T3SS substrates and nonsecreted proteins. The dashed line represents the negative (background) training set, whereas the dotted line represents the T3SS substrate set. The estimates were calculated for residues 2–47 using a sliding window size of 3.

To compare the composition of T3SS substrates to other nonsecreted proteins, we used an information theory-based classifier that involves computations of relative entropy [49], [50]. This classifier analyzed the T3SS substrate and background training sets using a sliding window size of 3. For each window block, a probability score (or entropy estimate) was calculated as described in the Materials and Methods. For the T3SS substrate sequences, the entropy estimate for each sliding window was fairly constant at about 4.1 bits (Figure 4). In contrast, the background data set differed in information content from the T3SS substrate set by between 0.1 to 0.5 bits. This result, along with findings from others, confirms that differences in amino acid composition can be exploited to develop computational models that recognize T3SS substrates [38], [51].

### Classification of DC3000 T3SS substrates based on relative entropy measurements

To distinguish DC3000 T3SS substrates from other proteins, we developed an algorithm incorporating a symmetric version of the Kullback-Liebler distance. The classifier, which we named the TEREE (Type III Effector Relative Entropy Evaluation) algorithm, was trained on the DC3000 T3SS substrate and background data sets. The algorithm was then used to evaluate all annotated protein coding sequences in the *P. s.* tomato DC3000 genome. Each protein received a relative entropy score between −47 and +34. All T3SS substrates that were used to construct the T3SS substrate training set scored between −47 and −11 (Table 2 and Table S1). Classifier performance was tested by constructing a negative training set of proteins known not to be secreted by the T3SS. Table S2 shows the score distribution for the supervised performance test. Based upon this table, we chose −13 as the cut-off score for predicting T3SS substrates. For blind classification tests involving the complete genome, Table 2 and Table S1 indicate that all but one protein in the substrate training set (HopAI1) had a score below (more negative than) −13.

In addition to the proteins in the substrate training set, the TEREE algorithm classified several other DC3000 proteins as potential T3SS substrates. These proteins, which scored between −47 and −13, fell into three classes: i) experimentally validated T3SS substrates that were omitted from the substrate training set, (ii) predicted substrates of the flagellar T3SS, or (iii) unlikely T3SS substrates. Proteins in the first class included HopD, HopO1-3, HopP1, HopS1, HopT1-2, HopAA1-2, HopAG1, HopAH2-1, HopAM1-2, HopAO1, HopAQ1, HopAS1, and PSPTO_0907 (Table 2). The fact that these omitted effectors earned scores similar to proteins in the T3SS substrate training set showed that the TEREE algorithm effectively identified DC3000 effector proteins. In fact, only one known DC3000 T3SS substrate omitted from the substrate training set, HopAH2-2, did not score within the −47 to −13 range. Proteins in the second class included FliC (flagellin), FlgM, FliK, FlgE (two homologs), and FlgK (Table S1). These results were not surprising, because flagellin can be secreted by nonflagellar T3SSs in other bacteria, and effectors can also be secreted through the flagellum [24], [52]–[54]. Finally, TEREE identified 63 proteins in the third class. We classified these proteins as unlikely T3SS substrates because they have predicted functions in bacterial cell physiology, metabolism, or transcription regulation. Furthermore, none of the genes encoding these proteins are regulated by HrpL, an extracytoplasmic function (ECF) family sigma factor that induces expression of almost all T3SS substrates in DC3000 [13], [41], [55], [56].

To further evaluate the effectiveness of TEREE, we performed several statistical tests on the results. First, we measured the sensitivity, which determines how accurately the algorithm identifies known T3SS substrates. At the cutoff score of −13, the sensitivity of the TEREE algorithm was 96.2%. This value is comparable to or better than the sensitivities achieved by other T3SS substrate predictive models [38], [43], [51], [57], [58]. Second, we determined the specificity, which assesses the proportion of proteins that are correctly identified as non- substrates of the T3SS. The specificity was 98.9%, which indicates that only about 1% of the proteins encoded by the DC3000 genome were incorrectly identified by TEREE as T3SS substrates. This value is significantly higher than the specificity values reported by most other computational models [38], [43], [51], [57], [58]. We also constructed a receiver operator characteristic (ROC) curve by plotting the sensitivity versus the specificity at each score output of the TEREE algorithm and calculated the area under the curve (AUC). The AUC measures the overall effectiveness of the algorithm at predicting T3SS substrates; a value of 1.0 indicates that all proteins were categorized correctly, whereas a value of 0.5 indicates that all proteins were randomly classified. The AUC for the TEREE algorithm was .992, indicating that it is highly accurate. Finally, TEREE performance was evaluated by a 5-fold cross validation test, in which 7–8 different effectors were randomly omitted from the positive training set in 5 distinct repetitions. The average sensitivity for the 5-fold cross validation was 90%, whereas the average specificity was 99.1% (data not shown). Therefore, the TEREE algorithm retained its predictive value in identifying DC3000 T3SS substrates even when the positive training set was varied.

### TEREE algorithm performance on other bacterial genomes

The universality of TEREE was evaluated by conducting analyses on other bacterial genomes that encode T3SSs. In each case, the algorithm was trained on the T3SS substrate and background data sets from DC3000. First, we examined *P. syringae* pathovar phaseolicola (*P. s.* phaseolicola) strain 1448a, which is closely related to *P. s.* tomato DC3000, but has a different host range. Although *P. s.* tomato DC3000 and *P. s.* phaseolicola 1448a encode many homologous effectors, they also each express several distinct effectors [6], [13], [42], [45]. TEREE identified 78.1% of the known T3SS substrates in 1448a and had a specificity of 98.7% (Table 3 and Table S3). In addition, the PSPPH_1525 and PSPPH_A0133 proteins were classified by TEREE as potential T3SS substrates. These proteins are likely to be effectors because they are both: i) encoded by genes that are regulated by HrpL [42], and ii) homologous to SKWP2, a verified effector protein in *Ralstonia solanacearum* [59], [60]. When another T3SS computational SVM-based model called SIEVE (SVM-based Identification and Evaluation of Virulence Effectors) analyzed the 1448a genome, the results were more sensitive but less specific than those of the TEREE algorithm (Table 3) [43]. We also compared TEREE and SIEVE by determining the number of validated T3SS substrates within the top 50 scoring proteins. TEREE recognized 20 1448a T3SS substrates within the top 50 hits, whereas SIEVE identified only 9. Thus, TEREE is more accurate than SIEVE at recognizing effectors in a bacterium that is closely related to DC3000.

**Table 3:** Comparison of TEREE to other computational T3SS substrate prediction models.

Genome	Method	Sensitivity^a^	Specificity^b^	# of known T3SS substrates in top 50 hits
*P. s.* phaseolicola 1448a	TEREE	78.1%	98.7%	20
	SIEVE^c^	87.5%	90.1%	9
*S. e.* Typhimurium LT2	TEREE	20.5%	98.8%	8
	SIEVE^c^	86.4%	91.9%	9
*R. solanacearum* GMI1000	TEREE	50.0%	98.2%	28
	BPBAc^c^	63.9%	99.0%	42

a Values were calculated by dividing the number of validated effectors, or true positives, by the sum of the true positives and false negatives. The second columns of Tables S3, S4, and S5 list the validated effectors for *P. s.* phaseolicola 1448a, *S. e.* Typhimurium LT2, and *R. solanacearum* GMI1000, respectively.

b Values were calculated by dividing the number of true negatives (non-substrates of the T3SS) by the sum of the false positives and true negatives.

c The sensitivity and specificity values for SIEVE and BPBAc were calculated based on published data sets [38], [43].

TEREE was also used to identify effectors in a more distantly related bacterium, *Salmonella enterica* serovar Typhimurium (*S. e.* Typhimurium) strain LT2. Although *P. syringae* and *S. enterica* are both in the γ-Proteobacteria, *S. e.* Typhimurium is an animal pathogen that causes a typhoid-like disease in mice and gastroenteritis in humans. In addition, *P. s.* tomato DC3000 and *S. e.* Typhimurium LT2 do not appear to have any effector genes in common. When TEREE was used to identify T3SS substrates encoded by the LT2 genome, the sensitivity was 20.5% and the specificity was 98.8% (Table 3 and Table S4). In comparison, SIEVE recognized 86.4% of the LT2 T3SS substrates at a specificity of 91.9% [43]. When we lowered the specificity of TEREE to 90.9%, the sensitivity rose to only 47.7%. Thus, SIEVE outperforms TEREE on the *S. enterica* Typhimurium LT2 genome. However, both computational models identified a similar number of T3SS substrates within the top 50 highest scoring proteins (Table 3) [43].

TEREE performance was also assessed on the *Ralstonia solanacearum* GMI1000 genome. This bacterium is a plant pathogen in the β-Proteobacteria and a more distant phylogenetic relative of *P.syringae* than *S. enterica*. Although the *R. solanacearum* GMI1000 and *P. s.* tomato DC3000 genomes encode several homologous effectors, these plant pathogens also secrete many distinct effectors [6], [59]. Interestingly, the TEREE algorithm was more effective at recognizing T3SS substrates in *Ralstonia* than in *Salmonella*, generating a sensitivity of 50.0% and specificity of 98.2% (Table 3). Although the sensitivity may seem low, it is important to note that within the top 50 hits, TEREE identified 28 validated T3SS substrates, 2 putative T3SS substrates, and 2 secreted flagellar proteins (Table 3 and Table S5) [59]–[61]. In addition, TEREE identified more than 25 *R. solanacearum* GMI1000 effectors that do not have homologs in DC3000 (Table S5). Another SVM-based computational T3SS substrate prediction model called BPBAac performed somewhat better than TEREE, with a sensitivity of 63.8%, and a specificity of 99.0% (Table 3) [38]. BPBAac also identified 42 bona fide effectors within the top 50 hits of the algorithm [38]. Overall, these results indicate that TEREE performance is in many respects comparable to other computational T3SS substrate prediction methods.

### Analysis of a potential T3SS targeting signal in FtsX

Several of the proteins classified as T3SS substrates by TEREE are not likely effector proteins because they have known or predicted intracellular functions. An example of such a protein is FtsX, the transmembrane component of an ABC transporter involved in cell division [62]. This protein was classified as a T3SS substrate by the TEREE algorithm in *P. s.* tomato DC3000, *P. s.* phaseolicola 1448a, and *S. e.* Typhimurium (Tables S1, S3, and S4). We reasoned that the N-terminal region of FtsX might contain functional T3SS targeting signals, while other features of the protein might prevent secretion. For example, the TMpred program (http://www.ch.embnet.org/software/TMPRED_form.html) estimates that FtsX contains four hydrophobic segments that span the cytoplasmic membrane. These membrane spanning regions might prevent FtsX secretion despite the presence of N-terminal T3SS targeting signals.

To determine whether FtsX contains T3SS targeting signals, we created an FtsX-Cya hybrid protein. According to TMpred, the N-terminus of the DC3000 FtsX protein is located in the bacterial cytoplasm and the first membrane spanning segment begins at amino acid 69. We thus removed all of the membrane spanning domains by fusing the first 50 amino acids of FtsX to Cya. This protein was expressed in wild-type or Δ*hrp* DC3000 strains, which were then inoculated into *N. benthamiana* leaves. As controls, we simultaneously measured the translocation of AvrPto_(1–164)_-Cya and AvrPto_Δ2–12_-Cya by DC3000. The AvrPto_Δ2–12_-Cya mutant lacks amino acids 2 to 12 of AvrPto, which removes most of the core signal required for targeting to the T3SS (Figure 1) [19]. Similar levels of cAMP were quantitated in *N. benthamiana* leaves inoculated with DC3000 strains expressing FtsX_(1–50)_-Cya or AvrPto_Δ2–12_-Cya, indicating that the N-terminal region of FtsX does not contain a functional T3SS targeting signal (Table 4). The lack of AvrPto_Δ2–12_-Cya and FtsX-Cya translocation was not due to poor protein expression or protein degradation, as both hybrids were detected in DC3000 (Figure 3). This finding highlights the importance of subjecting the results of computational prediction programs to experimental testing.

**Table 4:** Translocation of unlikely T3SS substrates into *N. benthamiana* by *P. s.* tomato DC3000.

Cya fusion protein	Translocation by DC3000 (pmol cAMP/µg protein)	
	Wild-type	Δ*hrp*
FtsX_(1–50)_	4.0±0.5	0.3±0.1
AvrPto_WT(1–164)_	155.4±23.5	0.5±0.1
AvrPto_Δ2–12_	7.5±1.0	0.1±0.0
AvrPto_FtsX(1–12)_	107.0±8.2	0.0±0.0
AvrPto_TccB(1–12)_	118.5±8.5	0.0±0.0

### The extreme N-termini of unlikely T3SS substrates do not prevent secretion of AvrPto

A number of studies on different T3SS substrates have shown that the minimal signal for targeting to the T3SS is located within the first 15 amino acids (or codons) of substrates [16]–[19], [21], [63]–[67]. We thus hypothesized that the extreme N-termini of nonsecreted proteins might prevent secretion of AvrPto. To test this idea, the first 12 amino acids of AvrPto_WT(1–164)_-Cya were replaced with the first 12 amino acids of FtsX to yield AvrPto_1–12FtsX_-Cya (Figure 1). Another similar fusion was constructed in which the first 12 amino acids of AvrPto were replaced with the same region of PSPTO_4342 (Figure 1). Because it is homologous to the TccB insecticidal toxin of *Photorhabdus luminescens*, we will refer to PSPTO_4342 as TccB. We predicted that the AvrPto_1–12TccB_-Cya fusion would not be translocated into plant cells for two reasons: i) TccB had a score of +18 in our computational model (Table S1), considerably outside of the range for T3SS substrates, and ii) a TccB-Cya fusion was not translocated into *N. benthamiana* by DC3000 in a previous study [41]. Both the AvrPto_1–12FtsX_-Cya and AvrPto_1–12TccB_-Cya hybrid proteins were efficiently expressed in DC3000 (Figure 3).

When the AvrPto-Cya hybrids with mutant N-termini were tested for translocation by the DC3000 T3SS into *N. benthamiana*, we unexpectedly observed that both the AvrPto_1–12FtsX_-Cya and AvrPto_1–12TccB_-Cya mutants were effectively delivered into plant cells in a T3SS-dependent manner (Table 4). The levels of cAMP that accumulated for each mutant were not much lower than that of the positive control, AvrPto_WT(1–164)_-Cya. Therefore, the minimal secretion signal of AvrPto appears to tolerate a number of substitutions. AvrPto is still a T3SS substrate even when its core secretion signal is replaced with sequences from proteins that are not translocated by the T3SS into host cells.

### Comparison of the abilities of computational models to accurately predict T3SS substrates

In addition to TEREE, SIEVE, and BPBAac, other computational models that predict T3SS substrates have been described [38], [43], [51], [57], [58], [68]. Because most of these programs are accessible as web-based prediction tools, we determined whether they could accurately classify the Cya hybrid proteins examined in this study as T3SS substrates. All of the computational models correctly predicted that wild-type AvrPto is a T3SS substrate, and that TccB is not secreted by the T3SS (Table 5). However, none of the models were able to successfully classify all of the other mutant AvrPto proteins. Interestingly, FtsX was predicted to be a T3SS substrate by three computational models other than ours, despite the fact that DC3000 was not able to translocate FtsX_(1–50)_-Cya into plant cells. Thus, computational tools may be helpful in identifying potential T3SS substrates, but the results can be misleading.

**Table 5:** Computational T3SS substrate predictions for proteins experimentally tested in this study.

Protein	Computational T3SS substrate predictions					Experimental results
	TEREE	SIEVE [36]	Effective T3^a^ [51]	T3SS prediction^b^ [57]	T3SEdb [68]	
AvrPto_WT_	+	+	+	+/+	+	+
AvrPto_SSM_	+	+	+/−	+/+	+	+
FtsX (PSPTO_0429)	+	−	+/−	+/−	+	−
TccB (PSPTO_4342)	−	−	−	−/−	−	−
AvrPto_Δ2–12_	+	−	+	+/+	+	−
AvrPto_FtsX(1–12)_	+	−	+	+/+	+	+
AvrPto_TccB(1–12)_	+	−	+	+/+	+	+

a This model could be run at more stringent (selective) or less stringent (sensitive) settings. Symbols in this column indicate that the protein was classified as a T3SS substrate at: (+) the selective level, (+/−) the sensitive level, or (−) neither level.

b This model could be run using either an ANN or SVM classifer. Symbols in this column indicate that the protein was classified as a T3SS substrate using: (+/−) ANN, (−/+) SVM, (+/+) both, or (−/−) neither classifiers.

## Discussion

Previous studies on AvrPto have shown that N-terminal amino acids are important for targeting to the T3SS. The first 15 amino acids of AvrPto are sufficient to target the Npt protein to the *Yersinia enterocolitica* T3SS for secretion into the extracellular milieu [19]. In DC3000, the first ∼50 amino acids of AvrPto are required for efficient secretion and translocation of an AvrPto-Cya hybrid protein [40]. AvrPto also possesses the characteristic N-terminal amino acid patterns associated with proteins traveling the T3SS pathway. The vast majority of actively deployed *P. s.* tomato T3SS substrates contain (i) greater than 10% serine, (ii) an aliphatic amino acid or proline at position 3 or 4, and (iii) no negatively charged residues within the first 12 amino acids [41]. However, some *P. syringae* effectors and many of the T3SS substrates from animal pathogens lack one or more of these characteristic patterns.

In this study, we tested the functional significance of the *P. syringae* T3SS targeting patterns in AvrPto. We found that AvrPto secretion into the extracellular milieu and translocation into plants was unaffected by multiple mutations that removed the three major patterns (Figure 2, Table 1). In fact, even though the first 15 amino acids of AvrPto are sufficient to target the Npt protein to the *Yersinia enterocolitica* T3SS for secretion into the culture medium, we found that replacing the first 12 amino acids of AvrPto-Cya with the same regions of the nonsecreted FtsX or TccB proteins did not appreciably reduce translocation into plant cells (Table 4). Therefore, instead of relying on a single targeting signal, AvrPto may have several characteristics that additively or redundantly contribute to its recognition by the T3SS. This model is consistent with our previous findings that secretion and translocation efficiency increases for AvrPto-Cya hybrids that contain progressively larger portions of AvrPto [40]. One feature of AvrPto that may play a role in recognition by the T3SS is a pH-folding switch controlled by histidine 87. This switch allows AvrPto to maintain an unfolded conformation in the bacterial cytoplasm [69]. Alternatively, AvrPto may interact with a chaperone that contributes to T3SS targeting. Another DC3000 effector, HopV, naturally lacks all three T3SS targeting patterns and interacts with the chaperone ShcV [70]. Thus, ShcV may compensate for a poor secretion signal by guiding HopV to the T3SS. However, there is currently no experimental evidence that chaperones mediate AvrPto secretion. Genes in the vicinity of *avrPto* do not possess features of T3SS chaperones, and promiscuous chaperones that interact with AvrPto have not been identified. In addition, AvrPto is secreted by *E. coli* containing a plasmid expressing the *hrp/hrc* T3SS gene cluster from *Dickeya dadantii* [71]. Thus, if AvrPto binds a chaperone, it is most likely encoded within the *hrp/hrc* gene cluster and conserved between *P. syringae* and *D. dadantii*.

Although our experimental analysis of T3SS secretion signals was limited to AvrPto, substantial changes have been made to the N-termini of several other effectors without radically reducing secretion. For example, AvrBs2 is delivered into plant cells by *Xanthomonas campestris* even when it contains frameshift mutations that alter the sequence of its first 18 amino acids [20]. Furthermore, YopE and YopD mutants that contain synthetic amphipathic amino acid sequences in their extreme N-termini are still secreted by the *Y. pseudotuberculosis* T3SS [22], [34], [67]. It has been proposed that substrate recognition by the T3SS is influenced by accessory proteins as well as the overall physical properties of substrates, rather than specific amino acid sequences [2]. Thus, it is possible that the AvrPto_1–12FtsX_-Cya and AvrPto_1–12TccB_-Cya hybrid proteins are translocated into plants because the FtsX or TccB amino acid sequences do not appreciably affect the structure of the AvrPto N-terminus.

To further examine compositional differences between DC3000 T3SS substrates and nonsecreted proteins, we employed a computational approach. According to our analysis, the amino acid sequences of T3SS targeting signals are substantially different than nonsecreted proteins (Figure 4). Other computational analyses have also recognized differences between the compositions of T3SS substrates and nonsecreted proteins [38], [51]. These differences were exploited to develop a computational algorithm based on a symmetric version of the Kullback-Liebler distance [50]. Unlike other computational T3SS substrate prediction algorithms that are based on SVM, ANN, or Naïve Bayesian classifiers, our method is based on information theory [38], [43], [51], [57], [58], [68]. The algorithm, called TEREE, distinguishes between T3SS substrates and other DC3000 proteins by calculating differences in relative entropy. The TEREE algorithm differentiated T3SS substrates in DC3000 with a high sensitivity; only two known effector proteins were not scored as positives (Table 2, Table S1). Another remarkable feature of TEREE is its high specificity. In other words, the majority of the top hits of the algorithm were known effectors, and only about 1% of the proteins in the DC3000 genome were scored as false positives.

Although TEREE performed extremely well in DC3000, its effectiveness in other bacteria varied (Table 3). The algorithm was efficient at recognizing effectors in *P. s.* phaseolicola 1448a and *R. solanacearum* GMI1000, but not in *S. e.* Typhimurium LT2. These results might be explained by the fact that *P. syringae* and *R. solanacearum* have several homologous effector genes [6], [59]. However, TEREE identified more than 25 *R. solanacearum* T3SS substrates that are not found in DC3000. Thus, the success of TEREE in *R. solanacearum* is not simply due to common effector genes. In contrast, *P. s.* tomato DC3000 and *S. e.* Typhimurium have different pathogenic lifestyles and completely distinct sets of effectors. Many *S. enterica* T3SS effectors function to promote bacterial entry into intestinal epithelial cells or survival within macrophages, while *P. syringae* effectors primarily suppress plant defense responses [72], [73]. TEREE performance on the *S. e.* Typhimurium genome thus might be improved by including *Salmonella* or other animal pathogen effectors in the T3SS substrate training set. Another reason that TEREE may not be as effective in *S. e.* Typhimurium is that *P. syringae* and *S. enterica* effectors have different amino acid biases. A recent analysis reported that plant pathogens contain more alanine, proline, and arginine in their effector targeting signals than animal pathogens [38]. In addition, animal pathogen effectors are more enriched in isoleucine, asparagine, and threonine than plant pathogen effectors [38]. Including animal pathogen effectors in the T3SS substrate training set for the TEREE algorithm might also compensate for this problem.

One false positive that was recognized as a T3SS substrate in several iterations of the TEREE algorithm was FtsX, a transmembrane protein that functions in cell division [62]. To explain these results, we reasoned that the N-terminal region of FtsX may possess a T3SS targeting signal that is obstructed by other features of the protein. In fact, when YopE is fused to a tightly folded protein such as dihydrofolate reductase (DHFR), it is rejected as a T3SS substrate [74], [75]. However, the first 50 amino acids of FtsX did not target the Cya reporter protein to the T3SS for translocation into plant cells (Table 4). Thus, even though the TEREE algorithm is quite sensitive, it does not rule out all nonsecreted proteins as T3SS substrates. TEREE is not alone in this regard. Several other computational T3SS substrate prediction programs were unable to precisely predict the secretion status of all the mutant AvrPto-Cya proteins examined in this study (Table 5).

In conclusion, advances in genome sequencing technologies have led to the availability of many new bacterial genome sequences. Computational T3SS substrate prediction models will be useful tools for identifying new effector genes within the genomes of bacteria that contain T3SSs. Our results show that the TEREE algorithm performed well on the genomes of three plant pathogens. No computational T3SS substrate prediction model is 100% accurate at identifying effector genes [38], [43], [51], [57], [58], [68]. Thus, comparing the results of a few different computational models and constructing a short-list of common hits may be the most effective way to identify potential T3SS effector candidates within bacterial genome sequences.

## Materials and Methods

### Bacterial strains and growth conditions

The *P. syringae* strains used in this study are listed in Table 6 and were grown in King's B medium (KB) at 29°C [76] or *hrp*-derepressing minimal medium supplemented with fructose (HDM) at 22°C [77]. *Escherichia coli* DH5α or TOP10 strains were used for cloning and propagating plasmids. They were grown in Luria-Bertani or Terrific Broth at 37°C [78]. Antibiotics were used at the following concentrations: ampicillin, 100 µg/ml; chloramphenicol, 20 µg/ml; gentamicin, 10 µg/ml; kanamycin, 50 µg/ml; rifampin, 50 µg/ml; spectinomycin, 50 µg/ml.

**Table 6:** Bacterial strains and plasmids used in this study.

Strain or Plasmid	Genotype or relevant characteristics^a^	Source
*E. coli*		
DH5α	F^−^ Φ80*lacZ*ΔM15 Δ(*lacZYA-argF*)U169 *deoR recA1endA1 hsdR17*(r_K_^−^ m_K_^+^) *phoA supE44 thi-1 gyrA96 relA1* λ^−^	Invitrogen
TOP10	F^−^ *mcrA* Δ(*mrr-hsdRMS-mcrBC*) Φ80*lacZ*ΔM15 Δ*lacX74 recA1 araD139* Δ(*ara-leu*)7697 *galE15 galK16 rpsL endA1 nupG*	Invitrogen
*P. syringae* pv. tomato		
DC3000	Wild type; Rf^r^	[83]
CUCPB5114	DC3000 Δ*hrpK-hrpR*::*ΩCm*; Rf^r^, Cm^r^	[48]
Plasmids		
pUFR034	Broad-host-range vector; Km^r^	[84]
pFLAG-CTC	Vector for expression of C-terminal FLAG fusion proteins; Ap^r^	Sigma-Aldrich
pCPP3156	pFLAG-CTC::*avrPto_Δ2–12_*	This work
pBBR1MCS-5	Broad-host-range expression vector containing P*_lac_*; Gm^r^	This work
pCPP3178	pBBR1MCS-5::*avrPto_Δ2–12_-FLAG*	This work
pCPP3384	pBBR1MCS-5::*avrPto_WT_*-*FLAG*	This work
pCPP3407	pBBR1MCS-5::*avrPto_SSM_*-*FLAG*	This work
pLMS153	pBBR1MCS-5::*avrPto*_2–12*ftsX*_-*FLAG*	This work
pLMS154	pBBR1MCS-5::*avrPto*_2–12*tccB*_ -*FLAG*	This work
pENTR/SD/D-TOPO	Gateway entry vector; Km^r^	Invitrogen
pCPP5168	pENTR/SD/D-TOPO::*ftsX*_(1–50)_	This work
pCPP3214	Vector for expression of C-terminal Cya fusion proteins; Sp^r^	[40]
pCPP3234	Gateway destination vector version of pCPP3214; Sp^r^, Cm^r^	[40]
pCPP5170	pCPP3234::*ftsX*_(1–50)_	This work
pND1	pCPP3214::*avrPto_SSM_*_ (1–50)_	This work
pND2	pCPP3214::*avrPto_SSM_*_(1–164)_	This work
pND3	pCPP3214::*avrPto_WT_*_ (1–50)_	This work
pND4	pCPP3214::*avrPto_WT_*_(1–164)_	This work
pLMS155	pCPP3214::*avrPto*_Δ2–12_	This work
pLMS157	pCPP3214::*avrPto*_2–12*ftsX*_	This work
pLMS158	pCPP3214::*avrPto*_2–12*tccB*_	This work

a Rf^r^, Cm^r^, Ap^r^, Gm^r^, Sp^r^, and Km^r^ indicate resistance to rifampicin, chloramphenicol, ampicillin, gentamicin, spectinomycin, and kanamycin, respectively.

### Construction of plasmids

pBBR1-based plasmids that express FLAG-tagged versions of wild-type and mutant AvrPto proteins were constructed in several steps. First, *avrPto* from *P. syringae* pv. tomato JL1065 was amplified by PCR using the primers P830C and P403C (Table 7). The product was digested with *Nde*I and *Sal*I and cloned into pFLAG-CTC. The resulting plasmid, pCPP3156, encodes an AvrPto protein that lacks amino acids 2–12 and contains a FLAG epitope (DYKDDDDK) at its C-terminus. The plasmid also contains a single point mutation that introduces an *Hpa*I cleavage site between codons 15 and 16 of *avrPto*, but does not change the amino acid sequence of AvrPto. The *avrPto_Δ2–12_-FLAG* sequence from pCPP3156 was then subcloned into pBBR1-MCS5 to create pCPP3178 (Table 6). To construct pCPP3384, which encodes AvrPto_WT_, pCPP3178 was digested with *Nde*I and *Hpa*I and ligated to a double-stranded DNA fragment formed by the hybridization of P831C and P832C (Table 7). Plasmids pLMS153 and pLMS154 were constructed in a similar manner, except that the double-stranded DNA fragments were formed by the hybridization P154 and P155, and P156 and P157, respectively (Table 7). To create pCPP3407, which expresses AvrPto_SSM_, pCPP3384 was digested with *Hpa*I and *Blp*I and ligated to a double-stranded DNA fragment that was formed by hybridizing four overlapping oligonucleotides designated APS1, APS2, APS3, and APS4 (Table 7). All oligonucleotides were phoshorylated by T4 polynucleotide kinase prior to hybridization.

**Table 7:** Oligonucleotides used in this study.

Name	Sequence^a^
P403C	5′-ATTGTAGTCGACTTGCCAGTTACGGTACGGG-3′
P830C	5′-GCGATACATATGCATCAGGTTAACTCCCCAGACCGAGT-3′
P831C	5′-TATGGGAAATATATGTGTCGGCGGATCCAGGATGGCCCATCAGGTT-3′
P832C	5′-AACCTGATGGGCCATCCTGGATCCGCCGACACATATATTTCCCA-3′
P154	5′-TATGAGTGCCACACGCAGCCCCAAGGTTTCAGAGCGCCATCAGGTT-3′
P155	5′-AACCTGATGGCGCTCTGAAACCTTGGGGCTGCGTGTGGCACTCA-3′
P156	5′-TATGTCCGATACCCTTGAAAGCCGGCTCAACGAATCTCATCAGGTT-3′
P157	5′-AACCTGATGAGATTCGTTGAGCCGGCTTTCAAGGGTATCGGACA -3′
APS1	5′- AACGCCCCAGACCGAGTTGCCAACAAC-3′
APS2	5′-GCGGGTGACGAAGATAACGTAACGGCCGCCCAACTGC-3′
APS3	5′-CTTCGTCACCCGCGTTGTTGGCAACTCGGTCTGGGGCGTT-3′
APS4	5′-TCAGCAGTTGGGCGGCCGTTACGTTAT-3′
P1	5′-CGGTTCTAGAACAATTTCACACAGGAG-3′
P2	5′-TAATATCCCGGGTGGTAGACCAGCAGACTC-3′
P3	5′-ATTTAACCCGGGTTGCCAGTTACGGTACG-3′
P1211C	5′-CACCATGAGTGCCACACGCA-3′
P1256C	5′-ATGACTCTCCAGCCAGGCAG-3′

a Important restriction enzyme sites are underlined.

The plasmids that express full length AvrPto_WT_, AvrPto_SSM_, AvrPto_Δ2–12_, AvrPto_2–12FtsX_, or AvrPto_2–12TccB_ fused to Cya (pND4, pND2, pLMS155, pLMS157, and pLMS158, respectively) were constructed in two steps. First, *avrPto* sequences were amplified from pCPP3178, pCPP3384, pCPP3407, pLMS153, or pLMS154 using the primers P1 and P3 (Table 7). Next, the PCR products were digested with *Xba*I and *Xma*I, and ligated to pCPP3214 digested with the same enzymes. The plasmids that express the first 50 amino acids of AvrPto_WT_ or AvrPto_SSM_ fused to Cya (pND3 and pND1, respectively) were constructed in a similar manner, except that P1 and P2 were used to amplify *avrPto* sequences from pCPP3384 or pCPP3407.

The plasmid that encodes the FtsX-Cya fusion protein (pCPP5170) was constructed using Gateway cloning technology (Invitrogen). *PSPTO_0429* sequences were amplified from DC3000 chromosomal DNA by PCR using P1211C and P1256C (Table 7). The PCR product was then cloned into pENTR/SD/D-TOPO to create the entry vector pCPP5168. A recombination (or LR) reaction between the entry vector and the destination vector pCPP3234 was then performed to create pCPP5170 (Table 6).

### DNA manipulations and sequencing

Plasmid DNA was isolated and manipulated according to standard protocols [79]. T4 polynucleotide kinase (New England Biolabs), restriction enzymes (New England Biolabs), and DNA ligase (Takara) were used according to the manufacturer's protocols. PCR was performed with either ExTaq (Takara) or Vent (New England Biolabs), and oligonucleotide primers were obtained from Integrated DNA Technologies (IDT). All cloned PCR products were sequenced to ensure that no mutations were introduced. DNA sequencing was performed at either the Cornell University Life Sciences Core Laboratories Center or the University of Missouri DNA Core Facility using an Applied Biosystems 3730 DNA analyzer (Applied Biosystems).

### Secretion assays, protein sample preparation, and immunoblot analysis

Secretion assays were carried out using a previously described procedure [40]. Cya hybrid protein expression from plasmids was monitored by inoculating *P. s.* tomato DC3000 strains into KB containing spectinomycin and 200 µM isopropyl-β-D-thiogalactopyranoside (IPTG). Cultures were grown at 28°C for 4 h, and bacteria were pelleted and suspended in protein sample buffer. Equal amounts of cells, based on OD_600_, were loaded onto an SDS-PAGE gel. Following separation by electrophoresis and transfer onto a nitrocellulose membrane, proteins were detected using a standard Western analysis procedure [79]. Primary antibodies, either anti-FLAG M2 mouse monoclonal immunoglobulin G (IgG) (Sigma-Aldrich), anti-Cya (3D1) mouse monoclonal IgG (Santa Cruz Biotechnology), or anti-NptII rabbit polyclonal IgG (United States Biological, Swampscott, MA), were used at 1∶5000. Secondary anti-mouse or anti-rabbit IgG-horseradish peroxidase conjugate antibodies (Sigma-Aldrich) were used at 1∶30,000. Blots were developed using the Pierce SuperSignal West Pico chemiluminescent substrate (Thermo Fisher Scientific).

### Adenylate cyclase assays

Cyclic AMP levels in infected *N. benthamiana* leaf tissue were determined as previously described [40], [80]. Briefly, *P. syringae* strains were grown as lawns on KB plates and then suspended to an OD_600_ of 0.3 (∼1×10^8^ cfu/ml) in 10 mM MgCl_2_-100 mM sucrose solution supplemented with 100 µM isopropyl-β-D-thiogalactopyranoside (IPTG). Bacteria were infiltrated into the third or fourth oldest leaves of *N. benthamiana* with a blunt syringe, and plants were incubated in a growth chamber set at 23°C and 80% humidity, with a 16 h/8 h light/dark cycle. Two leaf disks were collected from each infiltrated area 6 h post-inoculation with a 0.8-cm-diameter cork borer. Leaf disks were then frozen in liquid nitrogen, ground to a powder, and suspended in 300 µl of 0.1 M HCl. cAMP was quantitated using a cAMP ELISA assay kit (Enzo Life Sciences) and protein levels were determined by Bradford assay (Bio-Rad) according to the manufacturer's directions.

### Computational analysis of T3SS substrates

To characterize the composition of T3SS substrates, we divided the protein coding sequences of *P. s.* tomato DC3000 into two groups: i) a positive training set consisting of the amino acid sequences of 38 experimentally tested T3SS substrates (Table 2), and ii) the remaining ∼5600 protein sequences, which were used for background statistics. For the TEREE analysis, we extracted the first 50 amino acids of each sequence.

The block entropy calculation referred to in Figure 4 was accomplished by applying a sliding window to the positive training set and the background set. Let *W* represent the window size, N represent the number of amino acids (i.e. N = 20) and *M* represent the number of sequences in a given set. Under these circumstances, an *M×W* block of symbols was examined starting at sequence position *m* and ending at sequence position *m*+*W*−1. The block symbol probability at the m^th^ position, *p_i_*, was then estimated as *p_i_* = *n_i_/(MW)*, where *n_i_* is the number of times the i^th^ amino acid appears in the block for i = 1,…,N. The following equation was then used to determine entropy (*H_e_*) estimates for each window:
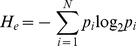
(1)


To identify T3SS substrates within bacterial genomes, the TEREE algorithm applies a symmetric version of the Kullback-Liebler distance [50]. Given two discrete probability mass functions P and Q each containing N elements, the symmetric Kullback-Liebler distance is defined as [49]:
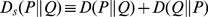
(2)where
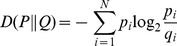
(3)is generally referred to as the relative entropy.

To characterize a protein sequence of unknown classification as being close or far from the substrate distribution, *D_s_* was evaluated over a series of sliding windows of length W. Given the window size, there were K = L-W+1 positions to consider where L = 50. At each window position, three discrete probability mass functions (Q, P^1^, P^2^) were computed: i) Q was constructed by computing *q_i_* = *n_i_/W* (*i* = 1,…,20) for the sequence of unknown classification, ii) *P^1^* represents the background probability mass function, and iii) *P^2^* represents the T3SS substrate probability mass function derived from *P. s.* tomato DC3000 sequences (Table 2). For the background and substrate distributions, similar to the block entropy calculation, we estimated the symbol probability as *p_i_* = *n_i_/(MW)*, where *n_i_* was the number of times amino acid *i* appeared in the window and M represents the number of sequences in a given set.

Given these distributions, the TEREE algorithm then calculated *D_s_(P^k^*|*Q)* for *k* = 1,2 where *P^1^* was the background distribution and *P^2^* was the T3SS substrate distribution derived from *P. s.* tomato DC3000 sequences (Table 2). Finally, category 2 was chosen if *D_s_(P^1^*∥*Q)*>*D_s_(P^2^*∥*Q)*; otherwise, category 1 was chosen. To decide upon the class membership of a given sequence, the choice for each of the K windows was examined and the majority was chosen. In other words, over *K* instances there were *k_1_* instances in favor of the background and *k_2_* instances in favor of the substrate distribution. A score *S* was created by taking the difference *S* = *k_1_−k_2_*. For the purposes of robustness, we ran our algorithm three times with window sizes *W* = 1,2,3. For each sequence tested, we took the minimum score from each of the three tests. All computations for this work were performed using MATLAB.

### Performance Evaluation

The performance of TEREE was evaluated by calculating three measures: i) sensitivity, or the number of true positives divided by the sum of the true positives and false negatives (TP/(TP+FN)), ii) specificity, or the number of true negatives divided by the sum of the false positives and true negatives (TN/(TN+FP)), and iii) the area under the ROC curve (AUC) that is generated when the sensitivity and specificity are plotted against each other for each output score. The AUC represents the probability that TEREE algorithm will rank a randomly chosen positive sequence at a score less than a randomly chosen negative sequence (Table S2). Specifically, the Wilcoxon Rank Sum Test was applied in order to compute the AUC [81], [82]. The 5-fold cross validation test was performed by creating 5 different T3SS substrate training sets that each lacked 7–8 different effector sequences. Each different training set was then utilized by TEREE to analyze DC3000 coding regions, and the sensitivity and specificity were calculated for each run.

## Supporting Information

Table S1 TEREE algorithm scores for annotated coding regions in *P. s.* tomato DC3000.
(XLSX)

Table S2 TEREE algorithm scores for proteins in T3SS substrate and negative training sets.
(XLSX)

Table S3 TEREE algorithm scores for annotated coding regions in *P. s.* phaseolicola 1448a.
(XLSX)

Table S4 TEREE algorithm scores for annotated coding regions in *S. e.* Typhimurium LT2.
(XLSX)

Table S5 TEREE algorithm scores for annotated coding regions in *R. solanacearum* GMI1000.
(XLSX)
